# Perceived difficulty of getting help to reduce or abstain from substances among sexual and gender minority men who have sex with men (SGMSM) and use methamphetamine during the early period of the COVID-19 pandemic

**DOI:** 10.1186/s13011-021-00425-3

**Published:** 2021-12-13

**Authors:** Kiffer Card, Madison McGuire, Jordan Bond-Gorr, Tribesty Nguyen, Gordon A. Wells, Karyn Fulcher, Graham Berlin, Nicole Pal, Mark Hull, Nathan J. Lachowsky

**Affiliations:** 1 School of Public Health and Social Policy, University of Victoria, Victoria, British Columbia Canada; 2 Canadian Institute for Substance Use Research, 291A Health and Wellness Building, Victoria, British Columbia V8P 5C2 Canada; 3 Faculty of Medicine, McGill University, Montreal, Quebec Canada; 4 Gay Men’s Sexual Health Alliance, Toronto, Ontario Canada; 5 Faculty of Medicine, University of British Columbia, Vancouver, British Columbia Canada; 6 Department of Psychology, Ryerson University, Toronto, Ontario Canada; 7 British Columbia Centre for Excellence in HIV/AIDS, Vancouver, British Columbia Canada

**Keywords:** Self-efficacy, Treatment barriers, Sexual and gender minorities, Methamphetamine

## Abstract

**Background:**

This study examined the perceived difficulty of getting help with substance use among sexual and gender minorities who have sex with men (SGMSM) who use methamphetamine during the early COVID-19 period.

**Methods:**

SGMSM, aged 18+, who reported sex with a man and methamphetamine use in the past 6 months were recruited to complete an online survey using online advertisements. Ordinal regression models examined predictors of greater perceived difficulty of getting help. Explanatory variables included participant characteristics (i.e., age, HIV status, ethnicity, sexuality, gender, region, income) and variables assessing patterns of methamphetamine use (i.e., frequency, % time methamphetamine is used alone and during sex; perceived need for help) and patterns of healthcare access (i.e., regular provider, past substance use service utilization).

**Results:**

Of 376 participants, most were gay-identified (76.6%), white (72.3%), cisgender (93.6%), and had annual incomes of less than $60,000 CAD (68.9%). Greater perceived difficulty of getting help was associated with having lower income, sometimes using methamphetamine prior to or during sex, and greater perceived need for help.

**Conclusion:**

Based on these results, we urge greater investments in one-stop, low-barrier, culturally-appropriate care for SGMSM who use methamphetamine. This is especially important given that participants who perceive themselves as needing help to reduce or abstain from substance use perceive the greatest difficulty of getting such help.

## Introduction

In Canada, less than 1% of the population used methamphetamine in the past year [1], with any lifetime use being approximately 4% [1]. Nonetheless, it is the third most commonly detected drug in illicit drug overdose deaths [2] – suggesting that people who use methamphetamine are in need of harm reduction and treatment services and supports. This is especially true for sexual and gender minority men who have sex with men (SGMSM), among whom the prevalence of methamphetamine is considerably higher. Estimates of methamphetamine use in the SGMSM population range from ten to twenty times greater than the general population [3]. For example, in a Vancouver-based sample of SGMSM recruited using respondent-driven sampling, an estimated 19.0% of participants used methamphetamine in the past 6 months, with 20.8% of those using at least weekly [4].

Two systematic reviews have evaluated the efficacy of methamphetamine treatment programs that aim to support SGMSM in changing methamphetamine use [5, 6]. These reviews highlight several potentially efficacious intervention strategies such as the use of cognitive behavioral, social support (e.g., support groups, social support strengthening), or other therapies [6]. However, the extent to which these interventions can benefit SGMSM is dependent on the person’s readiness to address their substance use, as well as the accessibility of effective supports and services and the ability of these supports and services to address interrelated health and social issues such as mental health problems and internalized homophobia that are strongly linked to substance use [5, 7, 8].

Not all SGMSM want to change their methamphetamine use [9, 10]. Literature examining methamphetamine use among SGMSM highlights its close association with “Party ‘n’ Play” (PnP) culture [11–13], which involves illicit drug use, especially methamphetamine, during sexual events. This can also include use of other drugs, such as amyl nitrate/butyrate (“poppers”), erectile drugs, gammahydroxybutyrate (GHB), and ketamine. The social connections that underlie the PnP scene, in addition to the biological drivers of methamphetamine dependence, can make it difficult for users to change their methamphetamine use [14, 15]; however, these social connections may also help individuals identify and access substance use supports and services [10, 16–18]. Matsuzaki et al. (2018) reports that people with greater social support have better perceived access to care and fewer barriers [19]. Likewise, membership in other social groups such as different income levels, age, ethnicity, region, education and gender can facilitate (or inhibit) the diffusion of health information (e.g., awareness of interventions) [20–22]. Seeing how perceived ability to access care is a common precondition to healthcare seeking and utilization [19, 23, 24], understanding these perceptions among SGMSM is critical to understanding interventions aimed to reduce harms among SGMSM who use methamphetamine.

In view of the importance of understanding the cascade of care for SGMSM who use methamphetamine, the purpose of this analysis was to explore factors associated with SGMSM’s perceived difficulty of getting help reducing or abstaining from substance use.

## Methods

### Context & Setting

In Canada, access to substance use services is patchwork, varying within and between provinces. This is true regarding both the cost and availability of substance use services. While general health services are covered by provincial health insurance programs, most substance use care falls under specialized programs. Few programs are available specifically tailored to SGMSM. Difficulty getting help was further compounded by the emergence of COVID-19 and associated lock downs in March 2021 – prior to which most of the data for this study were selected. Therefore, the context of COVID-19 must be considered in the interpretation of these findings.

### Participant recruitment

Participants were recruited between February 14th, 2020 and June 1st, 2020 using advertisements on geosocial networking applications (i.e., Squirt and Scruff) and social media posts (i.e., Facebook, Twitter, Reddit) shared by our study team and community-based organizations in British Columbia (e.g., Community-based Research Centre) and Ontario (e.g., Gay Men’s Sexual Health Alliance). Eligibility was restricted to men, transmen, and non-binary individuals (i.e. women were ineligible), who (1) were aged 18 years or older, (2) had sex with a man in the past 6 months, (3) used methamphetamine in the past 6 months, and (4) lived in Canada. Immediately before completing the survey online, participants gave informed consent and were screened for eligibility using an online survey. Participants received a $10 honorarium, paid by e-transfer or cheque as compensation for their time, which was approximately 30–45 min. This study was approved by the Research Ethics Board at Research Ethics BC, the University of Victoria, Simon Fraser University, and the University of British Columbia (REB #BC 17–485).

### Data collection

#### Survey development

The online survey was developed using a Community-Based Research approach that aims to address health inequities by engaging people with lived experience. This involved ongoing consultations with the SGMSM community throughout survey development including qualitative interviews with lived experiences with methamphetamine use to inform questionnaire items and structure and pilot testing.

#### Explanatory variables

Participants provided self-reported demographic information, information about their methamphetamine use, and their perceptions of healthcare access. Participant characteristics considered here included participant’s self-identified/reported age (in years), their ethnicity (which we collapsed into a binary variable representing white vs. non-white (self-reported) participants since most ethnicities were under-represented, creating small cell counts), gender (cisgender; transgender/non-binary), sexual orientation (grouped as gay vs. bisexual/other), self-reported HIV-status (I am HIV-positive; I think I am HIV-negative/I have never been tested for HIV), and province of residence (grouped due to some small cell counts as the Prairies [Alberta, Manitoba, and Saskatchewan], Eastern & Atlantic Canada [Ontario, Quebec, New Brunswick, Newfoundland & Labrador, Nova Scotia, Prince Edward Island]; Western Canada [British Columbia and Yukon Territory]).

Frequency of methamphetamine use was assessed over the previous 6 months (i.e., “Daily or almost daily”, “Weekly,” “Monthly,” “Once or twice,” “Never”). Participants who never used methamphetamine in the past 6 months were excluded from the study based on a priori inclusion criteria. Participants also reported the percent of the time they used methamphetamine prior to or during sex (what we operationalize as “sexualized methamphetamine use”) and the percent of time they used by themselves or alone (“Hardly any [0 - 19%];” “Only some [20 - 39%]”; “About half [40 - 59%];” “Most [60 - 79%];” “Nearly all [80 - 100%]”) over the previous 3 months.

Addressing issues related to healthcare access, participants indicated whether they had a primary healthcare provider, whether that provider knew they used methamphetamine, and whether they had accessed substance use services and supports in the past (“No,” “Yes, in the past 6 months;” “Yes, more than 6 months ago”). One item assessed whether participants felt they needed help with substance use (i.e., “In the past 6 months, to what extent did you feel you needed help in reducing your use of, or abstaining from (not using), substances?”). Responses were recorded on a 4-point ordinal scale ranging from “Completely” (1) to “Not at all” (4).

#### Outcome variable

The primary outcome of this analyses was a single question asking “At this time, overall, how easy is it for you to get help in reducing your use of, or abstaining from (not using) substances?” Responses were recorded on a 4-point ordinal scale ranging from “Completely” (getting help is completely easy) to “Not at all” (getting help is not at all easy).

### Data analysis

Statistical analyses were conducted in R Studio [25]. As a first step, we cleaned the data and excluded participants with any missing responses on the variables of interest. Bivariable differences in included and excluded participants were identified using binary logistic regression. This was done because observations with missing variables would not be included in multivariable models and we felt the demographic, bivariable, and multivariable data should all be analyzed based on the same underlying data. Differences between included and excluded participants were calculated. Descriptive and bivariable statistics were calculated using the CreateTableOne() function in the *TableOne* package and the polr() function [26].

The Brant test was used to test the proportional odds assumption given the ordinal outcome [27]. This test revealed that the proportional odds assumption held for the relationship with all explanatory variables included in the final model. Nevertheless, the ordinal outcome variable was modeled in two ways as a sensitivity test [27]: First a multivariable ordinal logistic regression model (predicting greater perceived difficulty of getting help) and, second, a binary logistic regression model (predicting “getting help is not at all [easy] vs. completely [easy]). Only variables with a *p*-value less than 0.20 in bivariate testing – indicating a relationship with our outcome – were included in final models. A comparison model with all variables was initially considered, but had poor statistical characteristics. A *p*-value of 0.05 was considered statistically significant.

## Results

**Fig. 1:**
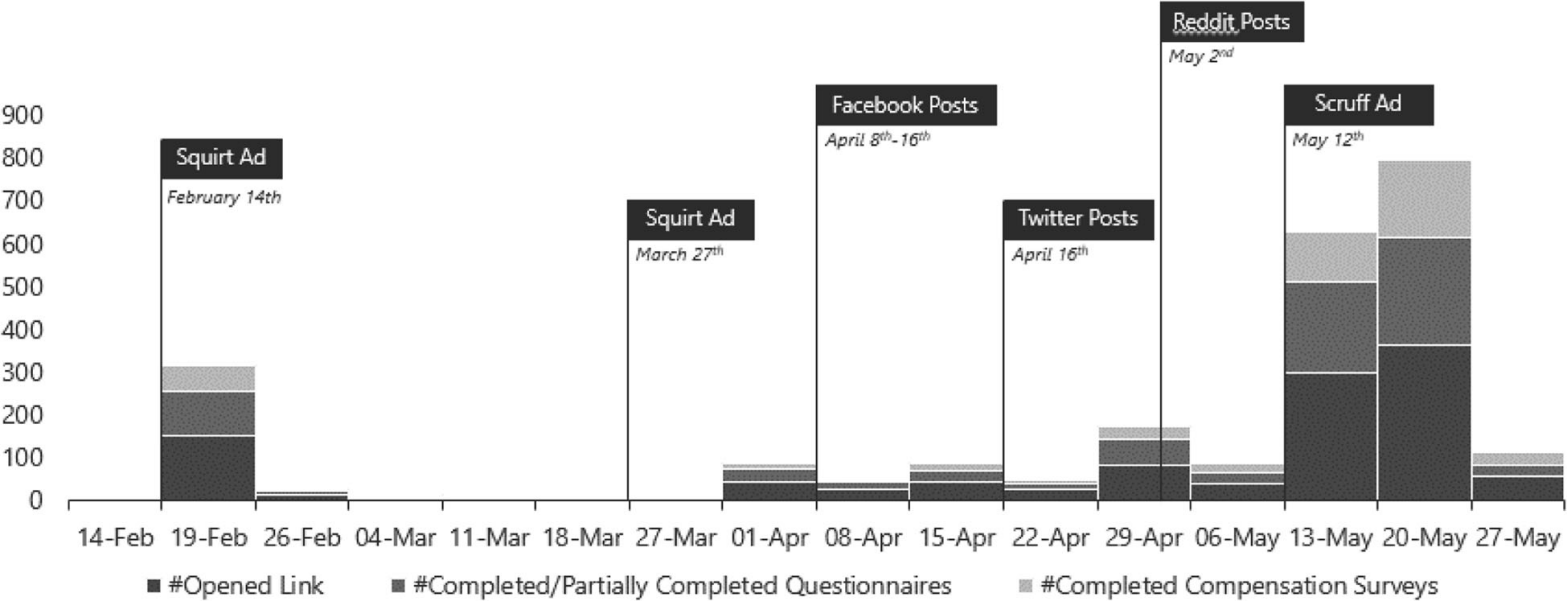
Recruitment timeline. Note: Most physicial distancing lockdowns were instituted in early March 2020 due to COVID-19

A total of 803 of those consented and were eligible to participate. Figure 1 shows the recruitment timeline, and illustrates that most participants were recruited following the institution of COVID-19 lockdown protocols in early March 2020. Due to high drop-off rates among participants, 376 observations were included in our analytic sample (15.2% dropped off before completing the demographics section [section 1], 33.5% before completing the substance use patterns section [section 2], and 44.5% before completing the healthcare access section [section 3]). Any participant with missing data was excluded from the study. Included and excluded participants did not differ based on age (*p* = 0.761), ethnicity (*p* = 0.990), gender (*p* = 0.822), sexual orientation (*p* = 0.154), income (*p* = 0.222), province of residence (*p* = 0.579), their frequency of methamphetamine use in past 6 months (daily, weekly, monthly, once or twice) (*p* = 0.059), whether they had ever received treatment/counselling/harm reduction services (*p* = 0.618), and whether they used methamphetamine before or during sex (0.916). Participants who were excluded were more likely to use methamphetamine ‘alone nearly all of the time’ vs. with others (24.1% vs. 14.1%; *p* = 0.024).

**Fig. 2:**
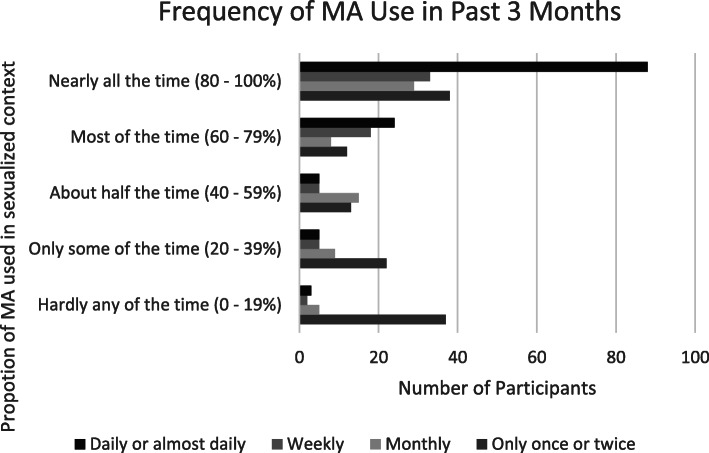
Ammount of time participants use methampehtamine before or during sex, by frequency of use in the past three months

Table 1 provides a demographic description of the analytic sample. Most participants were white (72.3%), gay (76.6%), cisgender (93.6%), and had annual incomes of less than $60,000 CAD (68.9%). Approximately one-third (33.2%) of participants reported using methamphetamine daily or almost daily and one-third (32.4%) reported using methamphetamine only once or twice in the past 6 months. The remainder reported using methamphetamine weekly (16.8%) or monthly (17.6%). Speaking to the often-sexualized nature of methamphetamine use, three-quarters of participants (76.6%) reported that for at least half of the time they were using methamphetamine, it was prior to or during sex. Half of the sample reported this sexualized context nearly all (80–100%) of the time (See Fig. 2). Greater frequency of methamphetamine use was also associated with a greater proportion of methamphetamine use within a sexual context (*p* < 0.0001).

**Table 1:** Sample Characterisitcs

	N (%)
**Age** *(mean (SD))*	*42.00 (11.75)*
**Person of Colour** (vs. White)	104 (27.7)
**Trans/Non-binary** (vs. Cisgender)	24 (6.4)
**Non-gay Identified**	88 (23.4)
**Region of Canada**	
Eastern & Atlantic Canada	199 (52.9)
The Prairies	46 (12.2)
Western Canada	131 (34.8)
**Living with HIV** (vs. HIV-negative/unknown)	129 (34.3)
**Never tested for HIV**	18 (4.8)
**Income**	
< $30,000	136 (36.2)
$30,000 - $59,999	123 (32.7)
$60,000 - $89,999	66 (17.6)
> $90,000	51 (13.6)
**Frequency of Methamphetamine use in Past Six Months**	
Daily or almost daily	125 (33.2)
Weekly	63 (16.8)
Monthly	66 (17.6)
Once or twice	122 (32.4)

**Fig. 3:**
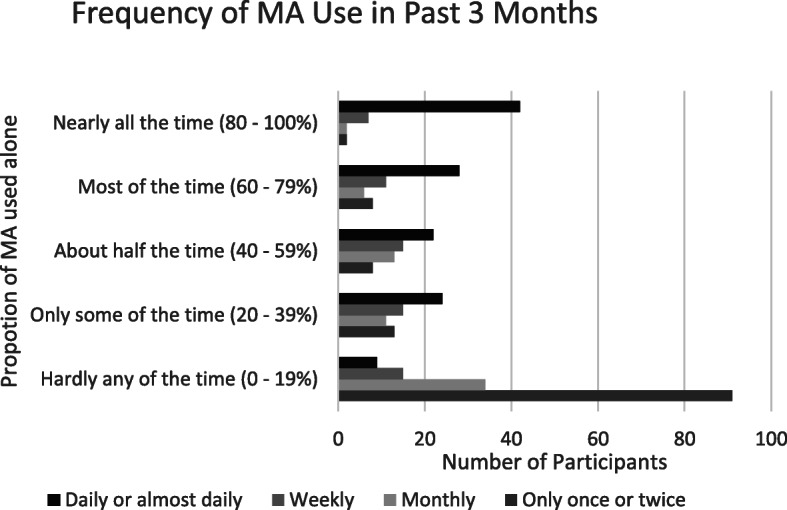
Ammount of time participants use methampehtamine alone, by frequency of use in the past three months

In regards to the social nature of methamphetamine use, 39.6% reported that they hardly ever used methamphetamine alone (See Fig. 3). A total of 14.1% reported using methamphetamine alone nearly all of the time and 15.4% reported using alone about half the time. A total of 33.6% of those who reported daily or almost daily use of methamphetamine reported using alone nearly all of the time; however, greater frequency of use was also associated with using alone more frequently (*p* < 0.0001).

Regarding mode of use in the past 6 months, 40.4% reported snorting methamphetamine and 23.3% reported injecting methamphetamine. Among participants who reported injecting methamphetamine, 21.7% used shared syringes, 27.9% used shared water, 20.9% used shared filters, and 19.4% used shared containers or spoons.

In terms of disclosure of methamphetamine use to a health care provider, 34.0% of the sample had a primary healthcare provider that was aware of their methamphetamine use, 43.4% had a primary healthcare provider who did not know about their use, and 22.6% did not have a primary healthcare provider at all. Among all participants, 6.1% felt they “completely” needed help reducing or abstaining from using substance, 17.6% felt like they needed help “a lot”, 41.8% felt like they needed “a little” help, and 34.6% felt they did “not at all” need help.

Table 2 shows bivariable comparisons for our analysis examining factors associated with perceived difficulty of getting help to reduce or abstain from substances. Greater frequency of methamphetamine use (*p* = 0.049), more frequent sexualized methamphetamine use (*p* = 0.003), and greater perceived need for help (*p* < 0.001) were associated with increased perceived difficulty of getting help. Lower income (*p* = 0.12) and not having a primary health care provider (*p* = 0.106) were marginally associated with increased perceived difficulty of getting help – but this association was not statistically significant. Contrary to our expectations, region of residence, past utilization of substance use services, and using methamphetamine alone were not associated with perceived difficulty of getting help to reduce or abstain from using substances.

**Table 2:** Bivariable Models of Perceived Ease of Access to Supports and Services

	At this time, overall, how easy is it for you to get help in reducing your use of, or abstaining from (not using) substances?				Bivariable
	1 - Completely	2	3	4 – Not at all	
	N (%)	N (%)	N (%)	N (%)	*p*-value
**Age** ***(mean (standard deviation))***	43.4 (11.7)	43.2 (11.8)	41.8 (12.3)	40.5 (9.9)	0.450
**Non-white (vs. White)**	20 (30.8)	18 (36.0)	51 (26.2)	15 (22.7)	0.380
**Trans/Nonbinary (vs. Cisgender)**	3 (4.6)	2 (4.0)	14 (7.2)	5 (7.6)	0.758
**Non-gay Identified**	8 (12.3)	10 (20.0)	25 (12.8)	7 (10.6)	0.863
**Person Living with HIV (vs. Not)**	20 (30.8)	22 (44.0)	62 (31.8)	25 (37.9)	0.339
**Region of Canada**					0.267
Eastern & Atlantic Canada	28 (43.1)	30 (60.0)	108 (55.4)	33 (50.0)	
The Prairies	12 (18.5)	2 (4.0)	24 (12.3)	8 (12.1)	
Western Canada	25 (38.5)	18 (36.0)	63 (32.3)	25 (37.9)	
**Income**					**0.120**
< $30,000	15 (23.1)	18 (36.0)	74 (37.9)	29 (43.9)	
$30,000 - $59,999	27 (41.5)	19 (38.0)	55 (28.2)	22 (33.3)	
$60,000 - $89,999	10 (15.4)	7 (14.0)	42 (21.5)	7 (10.6)	
> $90,000	13 (20.0)	6 (12.0)	24 (12.3)	8 (12.1)	
**Frequency of methamphetamine use in past six months**					**0.049**
Daily or almost daily	13 (20.0)	16 (32.0)	64 (32.8)	32 (48.5)	
Weekly	8 (12.3)	11 (22.0)	34 (17.4)	10 (15.2)	
Monthly	14 (21.5)	8 (16.0)	36 (18.5)	8 (12.1)	
Once or twice	30 (46.2)	15 (30.0)	61 (31.3)	16 (24.2)	
**MA use during or before sex**					**0.003**
Hardly any (0–19%)	19 (29.2)	4 (8.0)	20 (10.3)	4 (6.1)	
Only some (20–39%)	6 (9.2)	3 (6.0)	20 (10.3)	12 (18.2)	
About half (40–59%)	6 (9.2)	8 (16.0)	20 (10.3)	4 (6.1)	
Most (60–79%)	9 (13.8)	6 (12.0)	34 (17.4)	13 (19.7)	
Nearly all (80–100%)	25 (38.5)	29 (58.0)	101 (51.8)	33 (50.0)	
**MA use alone**					0.329
Hardly any (0–19%)	34 (52.3)	20 (40.0)	76 (39.0)	19 (28.8)	
Only some (20–39%)	5 (7.7)	10 (20.0)	33 (16.9)	15 (22.7)	
About half (40–59%)	10 (15.4)	9 (18.0)	31 (15.9)	8 (12.1)	
Most (60–79%)	8 (12.3)	7 (14.0)	26 (13.3)	12 (18.2)	
Nearly all (80–100%)	8 (12.3)	4 (8.0)	29 (14.9)	12 (18.2)	
**Healthcare Provider knows of methamphetamine use**					**0.106**
No regular provider	11 (16.9)	12 (24.0)	39 (20.0)	23 (34.8)	
Regular provider doesn’t know	33 (50.8)	17 (34.0)	90 (46.2)	23 (34.8)	
Regular Provider knows of methamphetamine use	21 (32.3)	21 (42.0)	66 (33.8)	20 (30.3)	
**Perceived Need for Help**					**< 0.001**
Not at all	37 (56.9)	20 (40.0)	56 (28.7)	17 (25.8)	
A little	23 (35.4)	16 (32.0)	98 (50.3)	20 (30.3)	
A lot	2 (3.1)	14 (28.0)	30 (15.4)	20 (30.3)	
Completely	3 (4.6)	0 (0.0)	11 (5.6)	9 (13.6)	
**Past Service Utilization**					0.851
No, never	45 (69.2)	30 (60.0)	133 (68.2)	41 (62.1)	
Yes, in the past 6 months	7 (10.8)	7 (14.0)	17 (8.7)	8 (12.1)	
Yes, more than 6 months	13 (20.0)	13 (26.0)	45 (23.1)	17 (25.8)	

Table 3 shows multivariable results of our analysis examining factors associated with perceived difficulty of getting help to reduce or abstain from substances. These results showed that greater perceived difficulty of getting help was associated with having lower income, more frequent sexualized methamphetamine use, and greater perceived need for help. Participants perceived it was easier to get help if they had a regular healthcare provider who knew about their methamphetamine use compared with those who had no healthcare provider. Individuals with a healthcare provider who did not know about their methamphetamine use were not statistically more or less likely to perceive greater difficulty of getting help. At the multivariable level, frequency of methamphetamine use in the past 6 months was not significant, though the direction of effects remained.

**Table 3:** Multivariable Model of Perceived Ease of Access to Supports and Services

	Ordinal Logistic	Binary Logistic
	aOR (95% CI)	aOR (95% CI)
**Income**		
< $30,000	1.00	
$30,000 - $59,999	**0.61 (0.38, 0.99)**	**0.54 (0.3, 0.95)**
$60,000 - $89,999	0.75 (0.43, 1.32)	0.94 (0.46, 1.96)
> $90,000	0.67 (0.35, 1.30)	0.61 (0.29, 1.31)
**Frequency of methamphetamine use in past six months**		
Once or Twice	1.00	
Monthly	0.95 (0.53, 1.73)	0.89 (0.45, 1.79)
Weekly	1.16 (0.62, 2.15)	1.02 (0.49, 2.11)
Daily or almost daily	1.71 (0.98, 3.01)	1.39 (0.71, 2.71)
**MA use during or before sex**		
Hardly any (0–19%)	1.00	1.00
Only some of the time (20–39%)	**4.42 (1.87, 10.56)**	**2.72 (1.03, 7.59)**
About half the time (40–59%)	1.78 (0.77, 4.14)	1.33 (0.51, 3.48)
Most of the time (60–79%)	**2.46 (1.12, 5.48)**	2.39 (0.97, 6.02)
Nearly all the time (80–100%)	**2.09 (1.06, 4.16)**	1.78 (0.83, 3.81)
**Healthcare Provider knows of methamphetamine use**		
No regular provider	1.00	1.00
Regular provider doesn’t know	0.72 (0.42, 1.20)	1.02 (0.54, 1.89)
Regular Provider knows of methamphetamine use	**0.49 (0.28, 0.84)**	0.65 (0.34, 1.23)
**To what extent did you feel you needed help in reducing your use of, or abstaining from (not using), substances?**		
Not at all	1.00	1.00
A little	1.55 (0.98, 2.45)	**2.09 (1.24, 3.56)**
A lot	**2.87 (1.58, 5.26)**	**2.21 (1.11, 4.55)**
Completely	**3.54 (1.41, 9.02)**	**4.14 (1.25, 18.9)**

## Discussion

### Primary findings

The present study sought to identify whether participant characteristics and patterns of methamphetamine use were associated with perceived difficulty getting help to reduce or abstain from using substances. Most data were collected after lockdowns were instituted across Canada to Control for COVID-19 transmission. Results showed that greater perceived difficulty in getting help to reduce or abstain from using substances during this time was more common among SGMSM with lower incomes, SGMSM who used methamphetamine within the context of sex, SGMSM with greater self-perceived need for help, and SGMSM without a primary care provider. Other demographic factors and previous utilization of substance use services and supports were not statistically associated with perceived difficulty of getting help to reduce or abstain from using substances.

### Implications

Each of our findings has important implications for addressing barriers to treatment among SGMSM. First, the finding that SGMSM who perceived a greater need for treatment also perceived a greater difficulty of getting help to reduce or abstain from using substances is highly concerning. It suggests that there are significant barriers among those who are most interested in changing their substance use. Those who have greater perceived need for help may have greater awareness of other barriers that may prevent them from accessing care, such as cost, stigma, and not being ready to stop using [28]. This may be compounded by internal dialogues of shame, guilt or embarrassment [29]. Conversely, those who don’t feel the need to adapt their drug use may not be seeking out support services and thus are unaware of whether or not they are hard to access. There is much room for improvement within the status quo public health and medical systems to better serve those seeking care. This is consistent with the significant amount of literature that highlights barriers to care for people who use drugs [30–34]. This challenge is all the more difficult given lack of consistent treatment guidelines and efficacious treatments for people who use methamphetamine, much less for populations with unique needs such as SGMSM. We recommend the development of consistent treatment guidelines, efficacious treatments, and public health messaging that supports engagement with marginalized populations, such as SGMSM.

Second, our finding that SGMSM who are lower income perceive greater difficulty in getting the help with their substance use echoes research findings examining low-incomes as a barrier to care [35–38]. Analyzing data from the Canadian Community Health Survey, Slaunwhite (2015) showed that individuals from low-income (<$29,999) households are significantly more likely to report all types of barriers to care [36]. These barriers to care among people living with low-income arise from the general living conditions of having a low-income, the poor quality of interactions with providers, and the complexity of the health system [35]. McCall et al. (2019) developed recommendations based on community-driven work to help address barriers to care for those who use drugs and are socially disadvantaged. These recommendations advocate for culturally sensitive care including engagement of those with lived experiences in care delivery, recognition amongst care providers of the layers of social inequalities individuals face and the importance of fostering an environment of trust, safety and respect (31). Implementing these recommendations alongside increased funding to support interdisciplinary, integrated services can help to reduce these barriers and streamline care (27). Other literature supports the creation of one-stop, low-barrier, integrated care that is culturally sensitive and trauma informed [39–43]. The need for these services is particularly important given the bifurcation of services tailored for SGMSM and other people who use drugs (i.e., SGMSM services may not be culturally safe to people who use methamphetamine and other services tailored for people who use methamphetamine may not be culturally safe to SGMSM [44]).

Third, our finding regarding sexualized methamphetamine use shows that SGMSM who participate in PnP culture face barriers to substance use supports access. Given that sexualized drug use is an important setting for social connectedness and sexual expression, participants may fear loss of social connection with their friends or loss of their sexual subculture and identity if they reduce or quit using methamphetamine [45]. It is important to note that sex is an important way for SGMSM to form social connections and friendships, and that PnP is a setting where this can occur, given the effects that drugs such as methamphetamine have on feelings of pleasure and connectedness [46]. Of course, these benefits do not necessarily negate harms may arise from PnP use. Indeed, we observed that greater frequency of use was associated with more frequent sexualized methamphetamine use. These deterrents in accessing care may be heightened by the stigmatization that exists between SGMSM services towards people who inject drugs (PWID) and vice versa [44]. This territorial stigmatization has been identified as a barrier to accessing healthcare. As a result, SGMSM who use methamphetamine may feel excluded from both services exacerbating inequalities in accessing support. It is essential that services that prioritize support for certain groups (e.g., for PWID or SGMSM) support and engage with each other to increase ease of access. This has implications for how support services are designed and located. Inclusive services that acknowledge the important role that sex plays in social connectedness for the SGMSM community may provide opportunities to address socially produced barriers to care.

Given the prevalence of injection drug use and sharing equipment in this sample harm reduction strategies should focus on providing harm reduction supplies and services for drug use and sexual activity in tandem, such as new needles, snorting kits, gloves, condoms, lubricant, HIV pre-exposure prophylaxis, and Hepatitis C screening. Organizations who provide these supplies and services may also be well placed to provide referrals to support services for reducing drug use. Initiatives that combine harm reduction strategies for both drug use and sexual health have been implemented successfully globally including in the United Kingdom, Germany, and Australia [47, 48]. Modelled after these programs, Canadian organizations such as MAX Ottawa’s campaign Spill the Tea, AIDS Community Care Montreal (ACCM)‘s Kontak program, and The Gay Men’s Sexual Health Alliance of Ontario (GMSH)‘s Party n Play Your Way are a few examples of organizations that are working to utilize peer driven engagement harm reduction strategies that can meet these service gaps.

Finally, we found that SGMSM without a regular primary healthcare provider (relative to those who had a doctor who knew about their methamphetamine use) were more likely to perceive getting help to reduce or abstain from using substances as difficult. Meanwhile, there was no difference between those who had a primary healthcare provider that did not know about their methamphetamine use and those who did not have a primary healthcare provider. Ensuring all people are attached to primary care providers may help ensure they receive referrals to substance use and mental health treatments [49]. Among patients that do have primary care providers, screening, brief interventions, and referrals to treatment can help open dialogues about substance use or patient education on treatment options and how to access the services and supports they want [49]. It is also important to note that patient-provider trust is crucial to ensuring that open dialogue is possible and for SGMSM to feel comfortable disclosing their methamphetamine use [50]. This can be done through proactive inquiry concerning sexual identity, gender identity, sexual behaviour, and routine monitoring of patient experiences, such as proactively asking for consent to discuss patient’s sexual and substance use history. Full-spectrum (e.g., harm reduction and treatment) and integrated care is also needed to support primary-care interventions [6, 51]. This includes services that go beyond traditional harm reduction models – such as supervised consumption sites – which may not be consistent with sexualized drug use. Indeed, given the central importance of social and sexual connection for SGMSM who use methamphetamine, services must allow for these sorts of community oriented interactions to occur without rigid, asexual, and beurocratic barriers to harm reduction. For example, pairing consumption sites with access to sexual health supplies and support services may help to foster a more inclusive and accessible care environment. Identifying and engaging patients who need access to primary care provider could also support substance use care for these individuals.

### Strengths and limitations

The present study is limited by its use of an online convenience sample and poor survey completion rates resulting in a modest sample size, however it is still within range of past online survey response and completion rates achieved with SGMSM and illicit drug use populations. Furthermore, our survey was designed prior to the start of COVID-19, however most responses were collected after the COVID-19 pandemic had already caused lockdowns and physical distancing. Further, our survey reached more SGMSM who used methamphetamine than what could be achieved through national surveillance programs, including Canada’s national behavioural surveillance study of SGMSM – The Sex Now Study – and the Canadian Community Health Survey. We also highlight that our recruitment strategy was further strengthened by consultations with the SGMSM community on appropriate and effective recruitment methods. While the survey used in this study has not yet undergone comprehensive psychometric validation, it has strong face and ecological validity. We note that some concepts (e.g., region of residence) could have been measured in ways that would have provided alternative results. For example, measuring rurality and urbanity may produce different results than measuring province/region of residence. As the survey development was informed by community-based methods including consultations with the SGMSM community the questions and structure reflect the sensitivities and attributes of the SGMSM and methamphetamine use context. Due to the self-reported nature of the study, potential response biases may exist, for example as the survey explored topics of drug use and sexual behavior participants may have provided socially desirable responses (e.g., reported less stigmatized use forms of methamphetamine use [52]). That said, given the novelty of this work within this population, our data point to future directions in research that could lead to more rigorous evaluation of the topics explored here. We hope that this work provides a foundational motive to generate higher quality data examining substance use services and support for SGMSM. We also note that the included and excluded participants differed (though not statistically) on important factors, including frequency of methamphetamine use. Efforts are needed to improve response and loss information bias, as the loss of these individuals could be an important factor in shaping statistical significance in bivariable and multivariable models.

## Conclusion

Our findings indicate that perceived difficulty of getting help abstaining or quitting from substances is elevated for those who are ready to make these changes, particularly so among those with low incomes, those connected to the PnP community, and those without a primary care provider. These findings highlight connection to care, income and social supports, and referral and linkage services as critical components of the treatment cascade for SGMSM who use methamphetamine. Better integrating health, social, and substance use support services may reduce some of the perceived difficulties of getting help for substance use among SGMSM.

## Data Availability

Data and materials for this study available upon request.

## Abbreviations

GHB: gammahydroxybutyrate (GHB)
SGMSM: Sexual and Gender Minority Men Who Have Sex With Men
PnP: Party and Play
